# Elevated acute phase proteins reflect peripheral inflammation and disease severity in patients with amyotrophic lateral sclerosis

**DOI:** 10.1038/s41598-020-72247-5

**Published:** 2020-09-17

**Authors:** David R. Beers, Weihua Zhao, Daniel W. Neal, Jason R. Thonhoff, Aaron D. Thome, Alireza Faridar, Shixiang Wen, Jinghong Wang, Stanley H. Appel

**Affiliations:** 1 Peggy and Gary Edwards ALS Laboratory, Department of Neurology, Houston Methodist Neurological Institute, Houston Methodist Research Institute, Houston Methodist Hospital, Houston, TX USA; 2 Department of Surgery, University of Florida College of Medicine, Gainesville, FL USA; 3 Department of Neurology, Houston Methodist Neurological Institute, 6560 Fannin Street, Suite ST-802, Houston, TX 77030 USA

**Keywords:** Immunology, Neurology

## Abstract

Amyotrophic lateral sclerosis (ALS) is a multifactorial, multisystem pro-inflammatory neuromuscular disorder compromising muscle function resulting in death. Neuroinflammation is known to accelerate disease progression and accentuate disease severity, but peripheral inflammatory processes are not well documented. Acute phase proteins (APPs), plasma proteins synthesized in the liver, are increased in response to inflammation. The objective of this study was to provide evidence for peripheral inflammation by examining levels of APPs, and their contribution to disease burden and progression rates. Levels of APPs, including soluble CD14 (sCD14), lipopolysaccharide binding protein (LBP), and C-reactive protein (CRP), were elevated in sera, and correlated positively with increased disease burden and faster progression. sCD14 was also elevated in patients’ CSF and urine. After a 3 year follow-up, 72% of the patients with sCD14 levels above the receiver operating characteristics cutoff were deceased whereas only 28% below the cutoff were deceased. Furthermore, disease onset sites were associated with disease progression rates and APP levels. These APPs were not elevated in sera of patients with Alzheimer’s Disease, frontotemporal dementia, or Parkinson’s Disease. These collective APPs accurately reflect disease burden, progression rates, and survival times, reinforcing the concept of ALS as a disorder with extensive systemic pro-inflammatory responses.

## Introduction

Amyotrophic lateral sclerosis (ALS) is the most common, devastating, and invariably fatal adult neuromuscular disease^1^. It is well established that neuroinflammation in the central nervous system (CNS) of patients with ALS is a prominent pathological observation and contributes to disease progression^2^. More recent evidence suggests that peripheral immune alterations/inflammation augment disease burden and rates of disease progression in ALS; ALS now is considered a multifactorial, multisystem disease in which the CNS and peripheral immune systems play important roles in development and progression of disease^2–7^. A recent meta-analysis reported that the levels of interleukins (IL-6 and IL-1β) and tumor necrosis factor-alpha (TNF-α), were increased in the blood of patients with ALS compared with healthy control (HC), implicating a peripheral systemic pro-inflammatory response^8^. Murdock et al. reported that early increased number of neutrophils and CD4 T lymphocytes in patients with ALS correlated with disease progression^9^. Gustafson et al. also reported that patients with ALS clustered into a profile distinct from HC primarily due to increased numbers of pro-inflammatory natural killer (NK)-like T lymphocytes and monocytes^10^. Another study reported that patients with ALS have chronic and persistent low-grade systemic inflammation that correlated with their degree of clinical disability and the production of specific proteins involved in inflammation; a heightened systemic inflammatory state is associated with a worse prognosis in ALS^11^. Thus, in addition to the well-known neuroinflammatory findings in patients with ALS, there is emerging evidence of clinically relevant peripheral immune pro-inflammatory responses in these patients.

The first reaction of the body to immunological stress is the innate, non-specific response preceding specific immune reactions^12^. The acute phase response (APR) is a prominent systemic reaction of the host to local or systemic disturbances caused by tissue trauma or inflammation, and within hours the pattern of protein synthesis by the liver is altered resulting in an increase of specific blood proteins, the acute phase proteins (APPs)^12^. The APR accompanies chronic as well as acute inflammatory states. Soluble CD14 (sCD14), lipopolysaccharide binding protein (LBP), and C-reactive protein (CRP), are known APPs^13–16^. Under the influence of IL-6, IL-1β, and TNF-α, hepatocytes synthesize and secrete APPs. Activation of circulating monocytes to a pro-inflammatory state induces the shedding of membrane bound CD14 (mCD14) to sCD14 and enhances the production of interleukins and TNF-α, possibly leading to synthesis and secretion of hepatic APPs^17–20^. In ALS, Keizman et al. reported that CRP is increased in blood of patients with ALS possibly due to a heightened peripheral inflammation^11^. A corroborating study found that patients with ALS that had elevated serum CRP levels progressed faster than those with lower CRP levels^16^. These studies, in addition to the well-known increased level of IL-6, IL-1β, and TNF-α in patients with ALS, suggest that peripheral immune alterations/inflammation cause increased hepatocytic synthesis and release of APPs that may augment disease burden, rates of disease progression, and survival times of patients with ALS.

Alterations of the peripheral immune system in patients with ALS should be interpreted in the context of the entire immune system and not just as single parameters. Therefore, in lieu of these data, there may be common mechanisms between liver expressed APPs and peripheral inflammation in patients with ALS; APPs may differentiate disease burden and rates of disease progression in patients with ALS. The objective of this study was to determine whether APPs were elevated in patients, and to discriminate disease burden and rates of disease progression^5,6^. This study also evaluated whether APPs were elevated in three other neurodegenerative diseases, Alzheimer’s Disease (AD), frontotemperal dementia (FTD) and Parkinson’s Disease (PD), and one autoimmune neurological disorder, chronic inflammatory demyelinating polyneuropathy (CIDP). Most prior studies have examined changes of individual proteins that might distinguish disease burden and rates of progression, but identification of a panel of APPs could increase the specificity and sensitivity of these clinical parameters^8,16,21,22^. This study provides evidence for a systemic pro-inflammatory immune response in the peripheral circulation of patients with ALS that is not observed in patients with AD, FTD, PD, or CIDP.

## Methods

### Monocyte isolation

Human monocytes were freshly isolated from peripheral blood of ALS patients and HC. A human pan monocyte isolation kit (Miltenyi Biotec, San Diego, CA, USA) was used to obtain highly pure pan monocytes by negative selection according to manufacturer’s instructions.

### Flow cytometry

Isolated pan monocytes were incubated with Fc blocker to avoid non-specific binding. Both isolated monocytes and fresh blood samples were then incubated with anti-human CD14-V450 antibody (eBioscience, San Diego, CA, USA), anti-human CD16-FITC (eBioscience, San Diego, CA, USA), anti-human HLA-DR-PerCP Cy5.5 (eBioscience, San Diego, CA, USA), anti-human TIM3-PE (eBioscience, San Diego, CA, USA). One additional lysing step to remove red blood cells was done for peripheral blood samples. Dead cells were stained using LIVE/DEAD Fixable Blue Dead Cell Stain Kit (Molecular Probes, Eugene, OR, USA). The cells were immediately analyzed using an LSR II™ 13 color flow cytometer configured with 355, 488, 405, 561, and 633 lasers.

### Quantitative RT-PCR

RNA samples were extracted and purified from monocytes using Direct-zol™ RNA MiniPrep Kit (Zymo Research) according to manufacturer’s recommendations. Quantitative RT-PCR was performed using one-step RT-PCR kit with SYBR Green (Bio-Rad Laboratories) and the iQ5 Multicolor Real-time PCR detection System (Bio-Rad Laboratories) according to manufacturer’s recommendations.

### ELISA

Human CD14 and CRP Quantikine, and LBP DuoSet, ELISA Kits from R&D Systems were used to determine the concentration of CD14, LBP, or CRP protein levels in patient and HC sera according to manufacturer’s instructions.

### Statistics

Comparisons were performed using ANOVA for more than 2 groups or Student's t-test for two groups. The ANOVA is presented with the degrees of freedom, F value, and *p* value. When a group had less that 30 measurements, a two-tailed Mann–Whitney U test was performed. The Mann–Whitney results are presented with a z score and a *p* value. Correlation was done using Spearman Rank Order in SigmaStat software. The Spearman correlation is presented with a rho (r) value and a *p* value. The Student’s t-test is presented with a *p* value. ROC curve analysis was performed using the R statistical software package (Vienna, Austria; V.3.0.2). Kruskal–Wallis test was used in the sites of disease onset. Data are expressed as mean ± SEM and *p* values less than 0.05 were considered significant.

### Study subjects

Standard protocol approvals, registrations, and patient consents were obtained. This was a prospective cohort study of patients with ALS and HC from our MDA/ALSA ALS clinic at the Houston Methodist Hospital. Written informed consent was obtained from all participants following ethics approval from the Institutional Review Board (IRB) at Houston Methodist Hospital. All studies were performed using ethical principles for medical research involving human subject in accord with the Declaration of Helsinki. Patients with ALS (recruited between January 2013 and November 2016) were diagnosed according to the revised El Escorial criteria and the Appel ALS (AALS) score (range 30–164) by an experienced ALS neurologist (SHA)^23,24^. Patients in the MDA/ALSA ALS clinic undergo detailed pulmonary function and nutritional assessments. All patients provided a detailed family history and were tested for common genetic mutations; i.e., superoxide dismutase 1 (SOD1), C9orf72, etc. Furthermore, all patients with ALS undergo detailed neuropsychological testing to identify possible cognitive impairments. Fast progressing patients were defined as those progressing at a rate of greater than or equal to 1.5 AALS points/month whereas slowly progressing patients progressed at less than 1.5 AALS points/month^3^. None of the patients with ALS had ongoing infectious diseases. HC were typically spouses and friends of patients, and exclusion criteria included any neurologic condition, autoimmune diseases, or infectious diseases. Clinical information was collected by the investigator from symptom onset and diagnosis to baseline assessment and sample collection. Two non-overlapping cohorts of patients and controls were used in this study—no patient or healthy control volunteer included in the first cohort was repeated/included in the second cohort. The demographics between the patients with ALS in the first cohort (n = 68) (mean [SD] age, 58.8 [1.57] years; 61.8% were men and 38.2% were women; and 90.7% were white, 4.65% were Hispanic, 2.33% were black, and 2.33% were Asian) and control individuals (n = 55) were similar (57.6 [2.15] years; 50.9% were men and 49.1% were women; and 90.9% were white, none were Hispanic, none were black, and 9.09% were Asian. In the second cohort (n = 100), the demographics of patients with ALS were 62.6 [1.47] years; 59.0% were men and 41.0% were women; and 85.9% were white, 4.56% were Hispanic, 6.03% were black, and 3.51% were Asian. Healthy control individuals in the second cohort (n = 60) were similar (63.5 [1.15] years; 45.0% were men and 55.0% were women; and 91.5% were white, 4.41 were Hispanic, none were black, and 4.09% were Asian. The demographics between the patients with dementia (n = 27) (75.2 [3.14] years; 63.0% were men and 37.0% were women) and control individuals (n = 13) were similar (76.9 [3.87] years; 46.2% were men and 53.8% were women).The demographics between the patients with CIDP (n = 14) (59.8 [3.14] years; 57.1% were men and 42.9% were women) and control individuals (n = 14) were similar (58.1 [5.20] years; 50.0% were men and 50.0% were women).The demographics between the patients with PD (n = 20) (71.1 [8.84] years; 60.0% were men and 40.0% were women) and control individuals (n = 20) were similar (68.3 [8.10] years; 30.0% were men and 70.0% were women).

### Human subjects

All studies were performed using ethical principles for medical research involving human subject in accord with the Declaration of Helsinki.

## Results

### Soluble CD14

**Figure 1:**
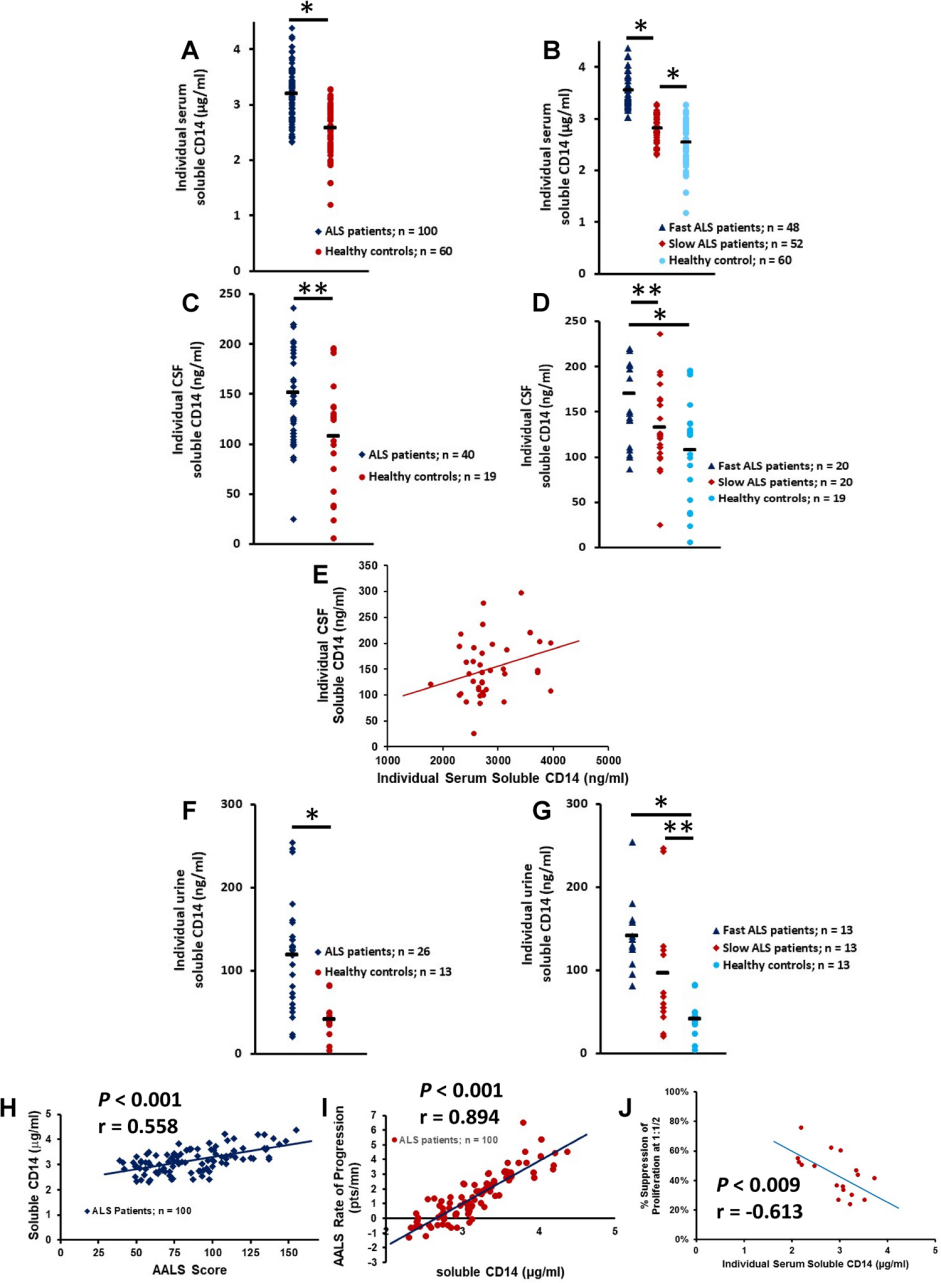
sCD14 is increased in patients with ALS and correlates with disease burden and disease progression. (**A**) In a cohort of patients with ALS (n = 100), serum sCD14 levels were elevated compared with HC (*p < 0.001). (**B**) When this cohort of patients was further separated into fast and slowly progressing patients, sCD14 was elevated in serum from fast (*p < 0.001) and slowly (*p < 0.001) progressing patients compared with HC. (**C**) sCD14 was elevated in the CSF of patients with ALS compared with CSF obtained from HC (**p = 0.020). (**D**) CSF samples from fast progressing patients had increased levels of sCD14 compared with slowly progressing patients (**p = 0.033) and with HC (*p = 0.002); there were no differences between slowly progressing patients and HC (*p* = 0.154). (**E**) sCD14 CSF levels positively correlated with serum levels of sCD14 (r = 0.315, *p* = 0.048). (**F**) sCD14 was elevated in the urine of patients with ALS compared with urine obtained from HC (*p < 0.001). (**G**) Fast and slowly progressing patients had increased urine sCD14 levels compared with urine sCD14 from HC (*p < 0.001 and **p = 0.022, respectively). (**H**) sCD14 levels were positively correlated with the patient’s burden of disease at the time of blood draw. (**I**) sCD14 levels positively correlated with the patient’s progression rate. (**J**) In a subset of patients with ALS, the patient’s sCD14 levels negatively correlated with their impaired Tregs suppressive function.

Hepatocytes are an important source of circulating sCD14, as sCD14 concentrations found in normal human blood exceed by 1 or 2 logs that of the cell mCD14^25^. In addition, upon monocyte activation, mCD14 is cleaved from the cell surface and sCD14 is released^20^. Thus, sera were examined for sCD14 levels. In the first cohort of patients (n = 68), serum sCD14 levels were elevated compared with serum from HC (*p* = 0.0001; n = 55) (Supplemental Fig. S1A). When further separated into fast progressing and slowly progressing patients, sCD14 was only elevated in serum from fast progressing patients compared with either slowly progressing patients (*p* < 0.001) or HC (*p* < 0.001) (*F*(2, 120) = 40.89, *p* < 0.0001) (Supplement Fig. S1B). There was no difference in serum sCD14 levels between slowly progressing patients and HC (*p* = 0.948). In a larger cohort of patients with ALS (n = 100), serum sCD14 levels were elevated compared with HC (*p* < 0.001; n = 60) (Fig. 1A), and were also elevated in serum from both fast (*p* < 0.001) and slowly progressing patients (*p* < 0.001) compared with HC (*F*(2, 157) = 109.68, *p* < 0.001) (Fig. 1B). There was no difference in the serum sCD14 levels in HC from the first and second cohorts (*p* = 0.736).

sCD14 levels were also evaluated in the CSF in a subpopulation of these patients and were tenfold less than that measured in the sera. CSF sCD14 was elevated in ALS patients compared with HC patients (z = 2.33, *p* = 0.020) (Fig. 1C). However, only CSF samples from fast progressing patients had increased levels of sCD14 compared with slowly progressing patients (*p* = 0.033) and HC (*p* = 0.002); there was no difference between slowly progressing patients and HC (*p* = 0.154) (*F*(2, 56) = 6.38, *p* = 0.0032) (Fig. 1D). Furthermore, CSF levels of sCD14 positively correlated with serum levels of sCD14 (r = 0.315, *p* = 0.048) (Fig. 1E).

Levels of urine sCD14 were also tenfold less than that measured in the sera. sCD14 was elevated in the urine of patients with ALS compared with urine obtained from HC (z = 3.89, *p* < 0.001) (Fig. 1F). When patients were separated into fast and slowly progressing patients, urine samples from fast progressing patients were not increased over urine levels of sCD14 in slowly progressing patients (*p* = 0.077). Fast and slowly progressing patients had increased urine sCD14 levels compared with urine sCD14 from HC (*p* < 0.001 and *p* = 0.022, respectively) (*F*(2, 36) = 12.38, *p* < 0.001) (Fig. 1G).

sCD14 can be generated either by cleavage from the cell surface or released from intracellular pools. To determine if CD14 mRNA is also decreased, qRT-PCR assays were performed on mRNA isolated from PBMC of patients and compared with mRNA isolated from HC. The CD14 mRNA was reduced in PBMC from patients (z = − 3.00, *p* = 0.003) (Supplemental Fig. S1C). When the mRNA expressions were analyzed in fast progressing and slowly progressing subpopulations, CD14 mRNA was only reduced in the fast progressing patients compared with either slowly progressing patients (z =  − 4.78, *p* < 0.001) or HC (z = 4.70, *p* < 0.001) (*F*(2, 74) = 19.09, *p* < 0.001) (Supplemental Fig. 1D). There was no difference in CD14 mRNA between slowly progressing patients with ALS and HC (z = 0.22, *p* = 0.826).

### sCD14 levels in patients with other neurological diseases

To determine the specificity of the increased ALS serum sCD14 levels, sera. from patients with dementia [Alzheimer’s disease (AD) and frontotemporal dementia (FTD)], were assayed for their sCD14 concentrations and compared with appropriate age-matched HC (n = 13). There were no differences in the serum levels of sCD14 from patients with dementia compared with HC (z = 1.27, *p* = 0.204) (Supplemental Fig. S1E). When patients were further separated into groups with mild cognitive impairments (MCI; n = 10), patients with AD (n = 13), and patients with FTD (n = 4), there were no differences among the different groups of patients (AD vs. MCI, z = 0.837, *p* = 0.401; MCI vs. FTD, *p* = 0.894; and AD vs. FTD, *p* = 0.742), and between each group of patients with dementia and HC (AD vs. HC, z = 1.69, *p* = 0.091; MCI vs. HC, z =  − 0.217, *p* = 0.826; and FTD vs. HC, *p* = 0.369) (*F*(3, 36) = 0.58, *p* = 0.630) (Supplemental Fig. S1F). Patients with dementia and their age-matched HC were not compared directly with patients with ALS and their respective age-matched HC because patients with dementia/HC were older than patients with ALS/HC (data not shown).

To determine if the increased serum sCD14 levels from patients with ALS were different from another neurological disease, serum from patients with chronic inflammatory demyelinating polyneuropathy (CIDP), an autoimmune neurological disorder, was assayed for their sCD14 levels and compared with appropriate age-matched HC. Serum sCD14 levels were not different between patients with CIDP and HC (z = 0.758, *p* = 0.447) (Supplemental Fig. S1G). In this analysis, the ages of patients with CIDP and their age-matched HC were not different than patients with ALS and their respective age-matched controls. Therefore, patients with CIDP were compared with patients with ALS (Supplemental Fig. S1H). In addition, since the ages between the two groups of HC were not different (z =  − 0.929, *p* = 0.751), the serum sCD14 levels between the two groups were compared. The two HC groups were then combined as no differences were detected (z =  − 0.647, *p* = 0.516). Serum sCD14 levels from all patients with ALS were increased compared with serum sCD14 levels from patients with CIDP (*p* = 0.042) as well as with the combined groups of HC (*p* < 0.001) (data not shown). When patients with ALS were separated into fast and slowly progressing patients, and then compared with patients with CIDP, only the fast progressing patients were different from patients with CIDP (z = 3.27, *p* = 0.001); no differences were found between slowly progressing patients and patients with CIDP (z =  − 0.542, *p* = 0.589) (*F*(3, 147) = 29.05, *p* < 0.001). There was no difference between patients with CIDP and the combined groups of HC (z =  − 1.39, *p* = 0.165).

Serum from patients with Parkinson’s disease (n = 20) was assayed for their sCD14 concentrations and compared with appropriate age-matched HC (n = 20). Serum sCD14 levels were not different between patients with PD and HC (z =  − 0.771, *p* = 0.441) (Supplemental Fig. S1I). Since the ages of patients with PD and their age-matched HC were not different than patients with ALS and their respective age-matched controls, serum sCD14 in patients with PD was compared with patients with ALS. Serum sCD14 levels from all patients with ALS were increased compared with serum sCD14 levels from patients with PD (z =  − 3.539, *p* < 0.001) (data not shown). The levels of serum sCD14 were not different between the two HC (z =  − 1.639, *p* = 0.101) (data not shown).

### Correlation between serum sCD14 and ALS progression rate and disease burden

Since sCD14 levels reflect the activation state of monocytes and subsequent pro-inflammatory responses, the correlation between serum sCD14 levels and increasing disease burden in patients with ALS based on the AALS scoring system was determined^23,24^. In the first cohort of patients, serum sCD14 levels were positively correlated with the patient’s burden of disease at the time of blood draw (*p* < 0.001, r = 0.684) (Supplemental Fig. S2A). In the second cohort of patients, serum sCD14 levels were again positively correlated with the patient’s burden of disease at the time of blood draw (*p* < 0.001, r = 0.558) (Fig. 1H). Also, in both patient cohorts, serum sCD14 levels were positively correlated with the patient’s progression rate (first cohort: *p* < 0.001, r = 0.727; second cohort: *p* < 0.001, r = 0.894) (Supplemental Fig. S2B, Fig. 1I, respectively). Further, since Tregs isolated from patients with ALS were shown to be dysfunctional^3,5^, the correlation of serum sCD14 levels with dysfunctional suppressive capabilities of ALS Tregs was determined in a subset of patients and a negative correlation was identified (*p* = 0.009, r = 0.613) (Fig. 1J).

### sCD14 predicts progression rates

**Figure 2:**
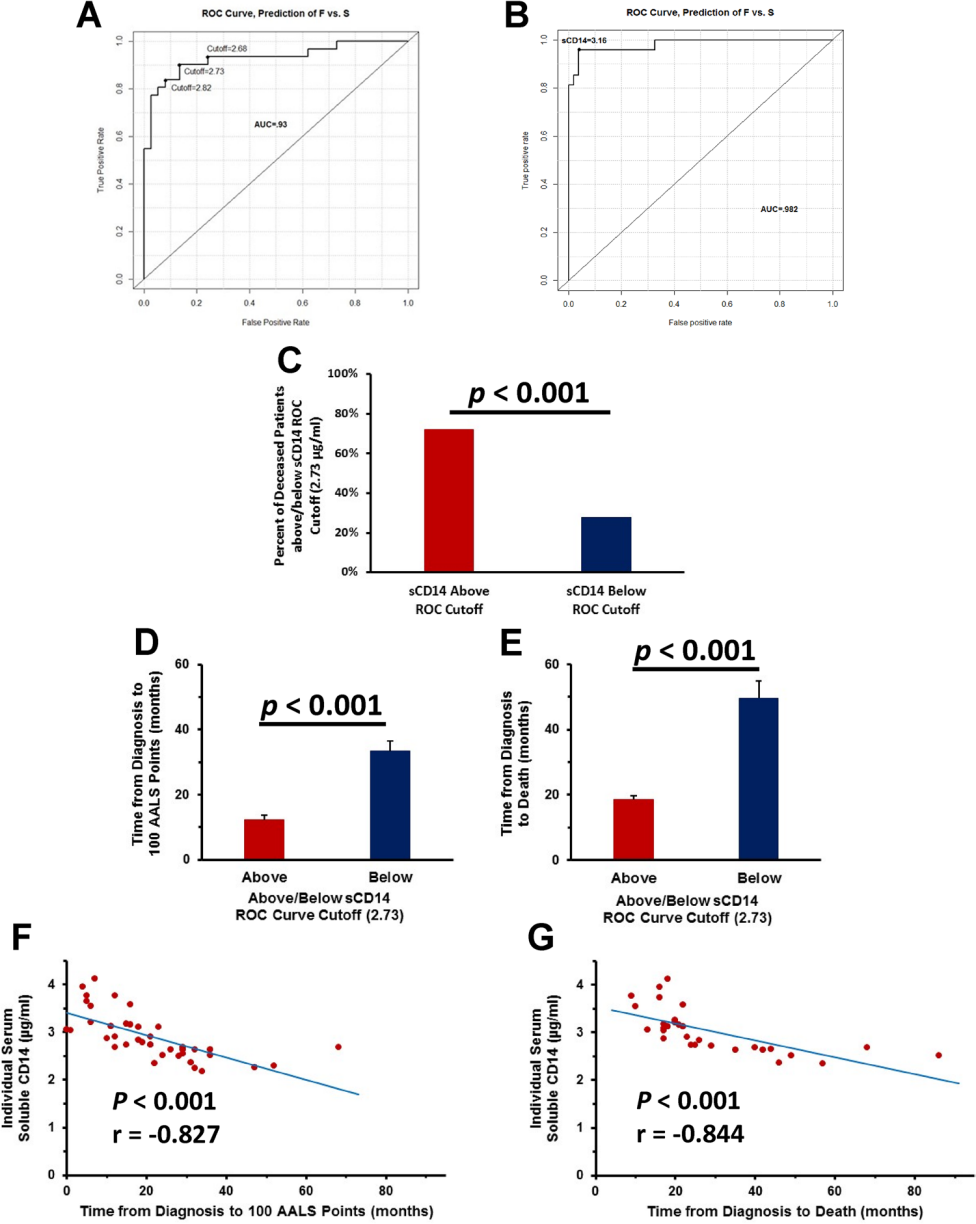
sCD14 levels accurately predict disease progression rates and survival times. (**A**) Serum sCD14 levels were an indicator of disease progression rates. (**B**) In a larger cohort of patients, serum sCD14 levels were an indicator of disease progression rates. (**C**) Seventy-two percent of patients who had sCD14 values above the cutoff were deceased while 28% of patients with sCD14 values below the cutoff were deceased. (**D**) Patients with a ROC score above the cutoff reached 100 AALS points faster than those patients with a score below the cutoff. (**E**) Patients with ROC scores above the cutoff lived a shorter period of time from diagnosis than those patients with scores below the cutoff. (**G**) The greater the amounts of serum sCD14, the faster the patients reached 100 AALS points. (**J**) The greater the amounts of serum sCD14 the faster the patients’ time from diagnosis to death.

The correlations between sCD14 and disease progression rates prompted the evaluation of the sCD14 serum levels as potential indicators of the patient’s current clinically assessed disease progression rate. Receiver operating characteristic (ROC) analyses were used to evaluate the accuracy of serum levels from these patients for differentiating fast versus slow disease progression rates at the time of serum collection^3^. In the first cohort of patients, serum sCD14 levels were an accurate indicator of disease progression rates. Using a ROC cutoff over 2.73 µg/ml of control as positive, serum sCD14 levels had a 90.9% accuracy, 88% sensitivity and 90% specificity for distinguishing fast progressing patients from slowly progressing patients (Fig. 2A). In the second cohort of patients, serum sCD14 levels were again an accurate indicator of disease progression rates. Using a ROC cutoff over 3.16 µg/ml of control as positive, serum sCD14 levels had a 98.2% accuracy, 94% sensitivity and 96% specificity for distinguishing fast progressing patients from slowly progressing patients (Fig. 2B).

At the end of this 3 year study with the first cohort of patients, 49% (33/68) of the patients were deceased while 51% (35/68) remained alive. Using the ROC score cutoff of 2.73 µg/ml of serum sCD14, 72% (23/32) of patients who had sCD14 values above the cutoff were deceased, while 28% (10/36) of patients with sCD14 values below the cutoff were deceased (χ^2^ = 13.9, *p* = 0.0003) (Fig. 2C). The patients with a ROC score above the cutoff reached 100 AALS points faster than those patients with a score below the cutoff (p < 0.001) (Fig. 2D). In accordance with the aforementioned findings, patients with ROC scores above the cutoff died within a shorter period of time from diagnosis than those patients with scores below the cutoff (p < 0.001) (Fig. 2E). Thus, the greater the amounts of serum sCD14, the faster the patients reached 100 AALS points and the faster the patients’ time from diagnosis to their deaths (*p* < 0.001, r =  − 0.827; *p* < 0.001, r =  − 0.844, respectively) (Fig. 2F,G).

### CD14 and CD16 surface markers on PBMC and isolated pan monocytes from patients with ALS and HC

Monocyte activation-dependent shedding of mCD14 is another source of sCD14; this activation leads to enhanced production of various cytokines, such as IL-1β and TNF-α^17–20^.

**Figure 3:**
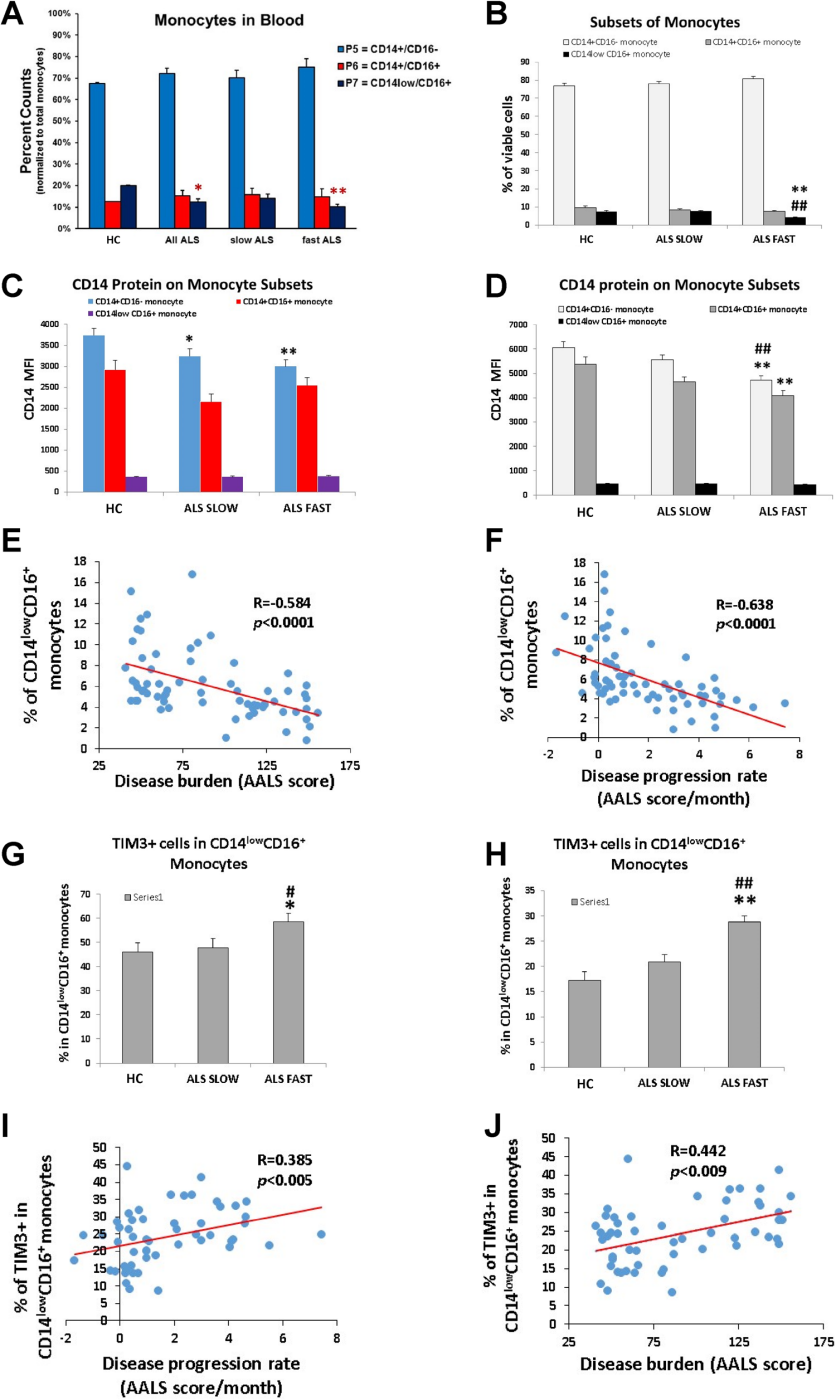
mCD14 is decreased on monocytes from patients with ALS and correlates with disease burden and disease progression rate. (**A**) CD14^−/low^/CD16^+^ monocytes were decreased in the total PBMC samples of all patients with ALS compared with HC (*p = 0.04). Fast progressing patients had reduced numbers of CD14^−/low^/CD16^+^ monocytes in their PBMC (**p = 0.016). (**B**) CD14^−/low^/CD16^+^ monocytes were decreased in fast progressing patients compared with slowly progressing patients (**p < 0.001) and HC (^##^*p* < 0.001). (**C**) Fast (**p < 0.01) and slowly (*p < 0.01) progressing ALS had reduced CD14 protein signal on surface of their CD14^+^/CD16^˗^ monocytes than HC. (**D**) CD14 MFI was decreased on CD14^+^/CD16^−^ and CD14^+^/CD16^+^ monocytes from patients with fast progressing ALS (**p < 0.01) compared with slowly progressing patients with ALS and HC (*p < 0.01). (**E**) CD14^−/low^/CD16^+^ monocytes were negatively correlated with disease burden. (**F**) CD14^−/low^/CD16^+^ monocytes were negatively correlated with rates of disease progression. (**G**) CD14^−/low^/CD16^+^/TIM-3^+^ monocytes were increased in PBMC of fast progressing patients compared with CD14^−/low^/CD16^+^/TIM-3^+^ monocytes from slowly progressing patients (^#^*p* < 0.001) and HC (*p < 0.001). (**H**) CD14^−/low^/CD16^+^/TIM-3^+^ monocytes were increased in fast progressing patients compared with slowly progressing patients (^##^*p* < 0.001) and HC (**p < 0.001). (**I**) CD14^−/low^/CD16^+^/TIM-3^+^ monocytes positively correlated with disease progression rates. (**J**) CD14^−/low^/CD16^+^/TIM-3^+^ monocytes positively correlated with burden of disease.

CD14 and CD16 are expressed predominately by monocytes/macrophages, and can be divided into three distinct subsets by flow cytometry: CD14^+^/CD16^−^, CD14^+^/CD16^+^, and CD14^−/low^/CD16^+^ monocytes. The frequency of CD14^−/low^/CD16^+^ monocytes was decreased in the PBMC samples of all patients with ALS compared with HC (*p* = 0.04) (Fig. 3A). Fast progressing patients had reduced numbers of CD14^−/low^/CD16^+^ monocytes in their PBMC compared with HC (*p* = 0.016). No differences were observed in the frequencies of the CD14^+^/CD16^-^ and CD14^+^/CD16^+^ populations.

In patients with ALS and age-matched HC, pan monocytes were isolated and purified from PBMC using a negative selection protocol to avoid possible monocyte activation, and then again, subjected to flow cytometric analyses; positive selection or lengthy gradient separation may activate monocytes. The frequency of the CD14^−/low^/CD16^+^ subset of purified monocytes was again decreased in fast progressing patients compared with slowly progressing patients (*p* < 0.001) and HC (*p* < 0.001) (Fig. 3B). As was observed in the PBMC samples, no differences were noted in the frequencies of the CD14^+^/CD16^−^ and CD14^+^/CD16^+^ populations.

To verify that cell surface expression of membrane-bound CD14 (mCD14) was indeed decreased, the CD14 median fluorescence intensity (MFI) was measured on the PBMC samples. Patients with fast (*p* < 0.01) or slowly progressing (*p* < 0.01) ALS had reduced CD14 protein signal on their CD14^+^/CD16^−^ monocytes compared with HC (Fig. 3C). Within the different monocyte subsets, the cell surface CD14 MFI was decreased on CD14^+^/CD16^−^ and CD14^+^/CD16^+^ monocytes from patients with fast progressing ALS compared with slowly progressing patients with ALS (*p* < 0.01) and HC (*p* < 0.01) (Fig. 3D). These data suggested that the frequency of CD14^−/low^/CD16^+^ monocytes was reduced in fast progressing patients with ALS, and the cell surface expression of CD14 on monocytes was reduced in both fast and slowly progressing patients.

### Correlation between CD14^−/low^/CD16^+^ monocytes and ALS disease burdens or rates of progression

The percentage of CD14^−/low^/CD16^+^ monocytes was negatively correlated with disease burden based on the AALS scoring system (*p* < 0.001, r =  − 0.584) (Fig. 3E); with greater disease burden, there was a reduced frequency of these monocytes. In addition, the percentage of CD14^−/low^/CD16^+^ monocytes was negatively correlated with rates of disease progression based on the AALS scoring system (*p* < 0.001, r =  − 0.638) (Fig. 3F); the faster the disease progression rate, the greater the reduction of these monocytes.

### CD14^−/low^/CD16^+^/TIM-3^+^subpopulation in CD14^−/low^/CD16^+^ cells in PBMC and isolated monocytes from patients with ALS and HC

TIM-3 has been shown to promote a pro-inflammatory response when expressed by innate immune cells^26,27^. The frequency of CD14^−/low^/CD16^+^/TIM-3^+^ monocytes was increased in PBMC of fast progressing patients with ALS compared with slowly progressing patients (^#^*p* < 0.001) and HC (*p* < 0.001) (Fig. 3G). The frequency of CD14^−/low^/CD16^+^/TIM-3^+^ isolated and purified monocytes was also increased in fast progressing patients compared with slowly progressing patients (*p* < 0.001) and HC (*p* < 0.001) (Fig. 3H) and was also positively correlated with disease progression rates and burden of disease (*p* = 0.005, r = 0.385; *p* = 0.009, r = 0.376, respectively) (Fig. 3I,J).

### Serum LBP concentrations

**Figure 4:**
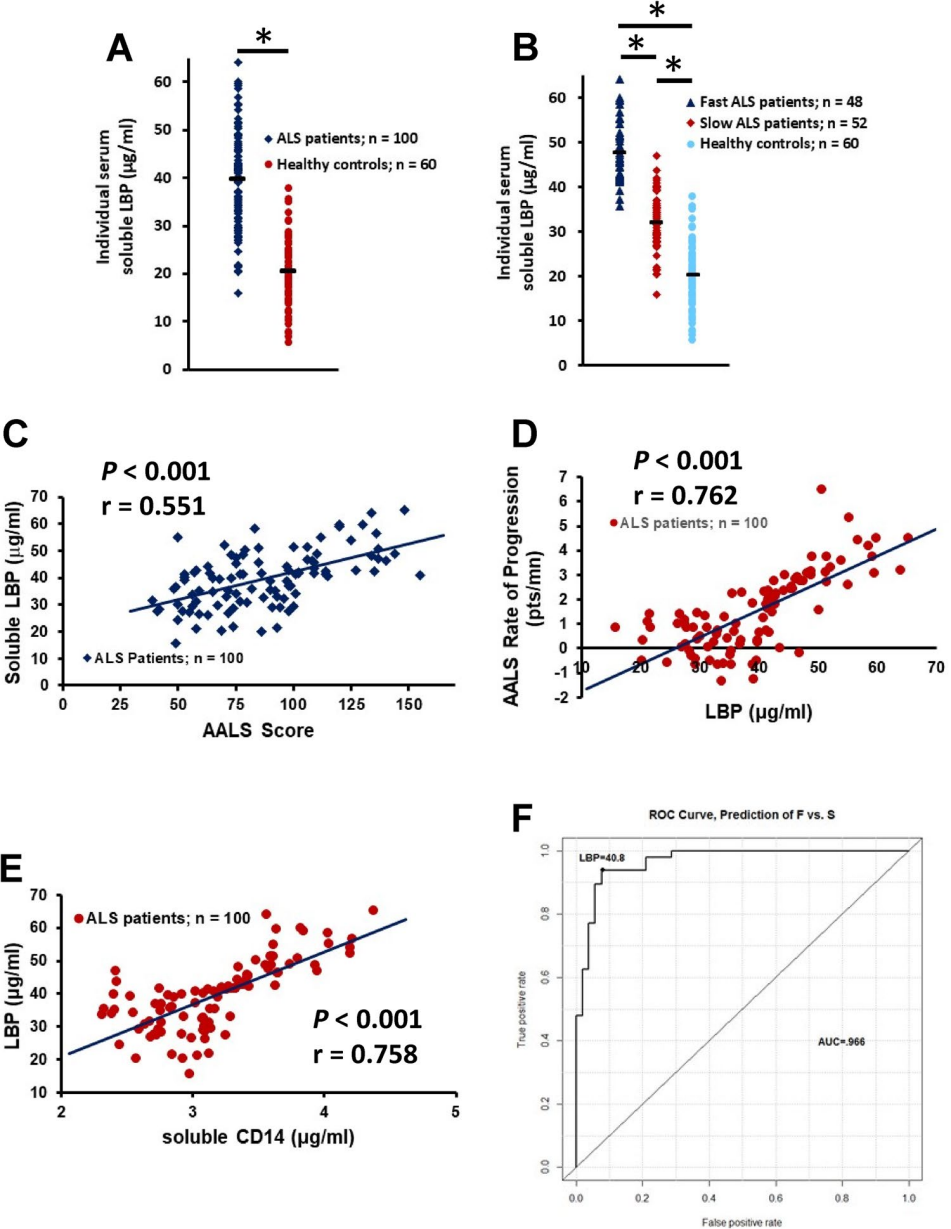
Serum LBP is increased in patients with ALS and correlates with disease burden and disease progression rate. (**A**) LBP was increased in the serum of all patients compared with HC (*p < 0.001). (**B**) LBP was elevated in fast (*p < 0.001) and slowly (*p < 0.001) progressing patients compared with HC. LBP was elevated in the fast progressing patients compared with slowly progressing patients (*p* < 0.001). (**C**) Serum LBP positively correlated with the patient’s burden of disease. (**D**) Serum LBP positively correlated with the patient’s disease progression rate. (**E**) There was a positive correlation between LBP and sCD14. (**F**) Serum LBP levels were an accurate indicator of disease progression rates.

Lipopolysaccharide binding protein (LPB) is a soluble acute phase protein that binds LPS. CD14 is a co-receptor for LPS, but can only bind LPS in the presence of LBP; binding of LPS and LBP to CD14 is necessary for signal transduction^13^. In the first cohort of patients, LBP was increased in the serum of all patients compared with HC (z = 3.79, *p* < 0.001) (Supplemental Fig. S2A), but was only elevated in fast progressing patients compared with either slowly progressing patients (z =  − 5.58, *p* < 0.001) or HC (z = 5.78, *p* < 0.001) (*F*(2, 75) = 51.52, *p* < 0.001) (Supplemental Fig. S2B). There was no difference in serum LBP between slowly progressing patients with ALS and HC (z = 1.21, *p* = 0.226). Although serum LPS/endotoxin was trending up in patients with ALS, the levels did not reach significance when compared with levels in serum from HC (data not shown). In the second cohort of patients, LBP was increased in the serum of all patients compared with HC (*p* < 0.001) (Fig. 4A), but was elevated in both fast and slowly progressing patients compared with HC (*p* < 0.001) (*F*(2, 157) = 201.60, *p* < 0.001) (Fig. 4B), although LBP was elevated in fast compared with slowly progressing patients (*p* < 0.001). There was no difference in the serum LBP levels between the HC in the first and second cohorts (*p* = 0.229).

Serum LBP was evaluated in patients with PD and was not elevated when compared with the HC (z =  − 1.555, *p* = 0.119) (Supplemental Fig. S1J). Since the ages of the HC for the patients with ALS and the HC for the patients with PD were not different, the levels of serum LBP were compared between patient groups. Serum LBP was increased in patients with ALS compared with patients with PD (z = 6.461, *p* < 0.001). There was not a difference in serum LBP between the HC for the patients and HC for the PD patients (z =  − 1.306, *p* = 0.190).

In the second cohort of patients, serum LBP positively correlated with the patient’s burden of disease (*p* < 0.001, r = 0.551) (Fig. 4C). Also in the second cohort of patients, serum LBP was positively correlated with the patient’s disease progression rate (*p* < 0.001, r = 0.762) (Fig. 4D). In addition, there was a positive correlation between LBP and sCD14 in the second cohort of patients (*p* < 0.001, r = 0.758) (Fig. 4E). Thus, increased LBP was accompanied by a concomitant increase in sCD14. In the second cohort of patients, serum LBP levels were an accurate indicator of disease progression rates. Using a ROC cutoff over 40.8 µg/ml of control as positive, serum LBP levels had a 93% accuracy, 93.8% sensitivity and 92.3% specificity for distinguishing fast progressing patients from slowly progressing patients (Fig. 4F).

### C reactive protein

**Figure 5:**
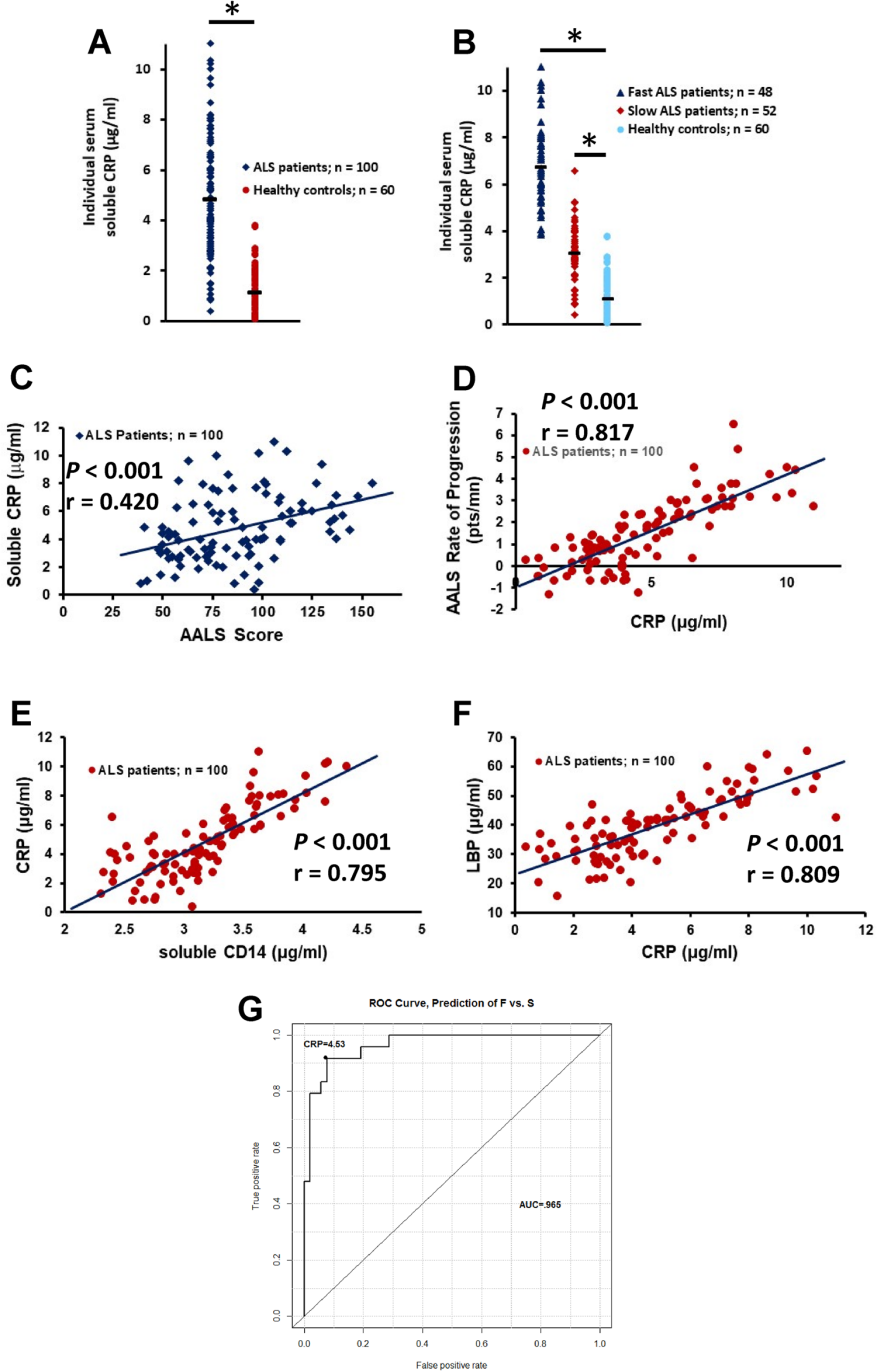
Serum CRP is increased in patients with ALS. (**A**) CRP was elevated in sera of patients compared with HC (*p < 0.001). (**B**) CRP was elevated in fast (*p < 0.001) and slowly progressing patients compared with HC. (**C**) Serum CRP was positively correlated with the patient’s burden of disease. (**D**) Serum CRP was positively correlated with the patient’s disease progression rate). (**E**) There was a positive correlation between CRP and sCD14 in patients. (**F**) There was a positive correlation between CRP and LBP in patients. (**G**) Serum CRP levels were an accurate indicator of disease progression rates.

A recent study demonstrated that serum C-reactive protein (CRP) was a prognostic biomarker in ALS^16^. Similar to sCD14 and LBP, CRP is produced by the liver and increases in the presence of inflammation. In a subpopulation of the first cohort, CRP was elevated in the sera of all patients compared with HC (z = 2.66, *p* = 0.008) (Supplemental Fig. S2C), but was only elevated in the fast progressing patients compared with either slowly progressing patients (z = 1.70, *p* < 0.048) or HC (z = 2.76, *p* = 0.006) (*F*(2, 27) = 8.17, *p* = 0.002) (Supplemental Fig. S2D). There was no difference in serum CRP between slowly progressing patients and HC (z = 1.78, *p* = 0.075). In the second larger cohort of patients, CRP was again elevated in the sera of all patients compared with HC (*p* < 0.001) (Fig. 5A) and also increased in both fast and slowly progressing patients compared with HC (*p* < 0.001) (*F*(2, 157) = 226.60, *p* < 0.001) (Fig. 5B). There were no differences in the serum CRP levels between HC in first and second cohorts (z = − 1.00, *p* = 0.317). CRP was not elevated in patients with CIPD (z = 0.069, *p* = 0.944) (Supplemental Fig. S2E).

Serum CRP was not elevated in PD (z =  − 1.609, *p* = 0.107) (Supplemental Fig. S1K). Since the ages of the HC for the patients with ALS and the HC for the patients with PD were compared; serum CRP was increased in patients with ALS compared with patients with PD (z = 5.764, *p* < 0.001). There was no difference in serum LBP between the HC for the patients and HC for the PD patients (z =  − 1.428, *p* = 0.153).

In the second cohort of patients, serum CRP was positively correlated with the patient’s burden of disease (*p* < 0.001, r = 0.420) (Fig. 5C). Also in the second cohort of patients, serum CRP was positively correlated with the patient’s disease progression rate (*p* < 0.001, r = 0.817) (Fig. 5D). In addition, there was a positive correlation between CRP and sCD14 in the second cohort of patients (*p* < 0.001, r = 0.795) (Fig. 5E). Furthermore, there was a positive correlation between CRP and LBP in the second cohort of patients (*p* < 0.001, r = 0.809) (Fig. 5F). Thus, as CRP increased there was a concomitant increase in both sCD14 and LBP. In the second cohort of patients, serum CRP levels were an accurate indicator of disease progression rates. Using a ROC cutoff over 4.53 µg/ml of control as positive, serum LBP levels had a 91% accuracy, 91.7% sensitivity and 90.4% specificity for distinguishing fast progressing patients from slowly progressing patients (Fig. 5G).

### Site of disease onset is associated with disease progression rates and APPs

Since disease burdens and disease progression rates positively correlated across all three APPs, all three APPs were compared with sites of disease onset. Table 1 shows that the site of disease onset was associated with rates of disease progression. Patients with bulbar onset more often exhibit faster rates of disease progression while patients with leg onset more often have slower rates of progression. Table 1 also shows no association between the age of the patient at symptom onset and the rate of disease progression.

**Table 1:** The association of disease onset sites with rates of disease progression.

	Fast (N = 48, 48.0%)	Slow (N = 52, 52.0%)	p-value (Fisher or Wilcox test)
**Onset site**			
Arm	24 (54.5)	20 (45.5)	
Leg	7 (25.0)	21 (75.0)	
Bulbar	17 (68.0)	8 (32.0)	
Other	0 (0)	3 (100)	0.002
**Age at first symp**			
Mean (SD)	58.4 (11.9)	58.7 (11.7)	
Median [IQR] (range)	59 (50, 67] (32.5, 80.9)	59 (52, 68] (30.2, 79.3)	0.948
Age at DX	60.5 (11.5)	60.7 (11.7)	
	61 (54, 69] (33.1, 82.5)	61 (54, 69] (32.2, 81.4)	0.947

Patients with bulbar onset exhibit faster rates of disease progression while patients with leg onset more often have slower rates of progression. There is no association between the age of the patient at symptom onset and the rate of disease progression.

*SD* standard deviation, *IQR* interquartile range, *DX* diagnosis.

Table 2 shows that the site of disease onset was associated with all three APPs. Patients with leg onset had lower APP levels, while patients with bulbar onset showed higher APP levels. Table 2 also shows that patients with arm onset were younger than those with other sites of disease onset. In addition, across all sites of disease onset, there was no correlation between the patients’ ages at diagnoses and progression rates.

**Table 2:** The association of disease onset sites with the APPs sCD14, LBP, and CRP.

	Arm (N = 44, 44.0%)	Leg (N = 28, 28.0%)	Bulbar (N = 25, 25.0%)	Other (N = 3, 3.0%)	p-value (Kruskal–Wallis test)
**sCD14**					
Median (range)	3.2 (2.3, 4.2)	2.9 (2.3, 4.2)	3.3 (2.4, 4.4)	3.1 (2.4, 3.1)	0.078
**LBP**					
Median (range)	41.3 (21.7, 59.8)	35.0 (20.2, 63.9)	42.7 (15.7, 65.2)	34.0 (31.3, 40.1)	0.097
**CRP**					
Median (range)	4.9 (0.85, 9.6)	3.8 (0.81, 11.0)	5.2 (0.38, 10.3)	4.1 (2.1, 4.2)	0.195
**Age at first symp**					
Median (range)	54 (30.2, 80.9)	61 (41.8, 77.0)	66 (32.5, 79.3)	61 (47.7, 63.3)	0.019
**Age at DX**					
Median (range)	58 (32.2, 82.5)	63 (42.9, 80.5)	67 (33.1, 81.4)	64 (52.1, 64.7)	0.038

Patients with leg onset had lower APP levels. In contrast, patients with bulbar onset had higher APP levels. Patients with arm onset were younger than those with other sites of disease onset. There was no correlation between the patients’ ages at diagnoses and progression rates.

*DX* diagnosis.

Since all three APPs in this study correlated with the patients’ disease progression rates, and since there was a correlation between disease onset sites and disease progression rates, the relationships between sCD14, LBP, and CRP levels and disease progression rates were determined. This analysis showed that overall onset site was associated with progression rates (*p* = 0.048), but only bulbar and leg onset sites were different in a head-to-head comparison (mean progression rate for bulbar = 2.1 AALS points per month, mean for leg = 1.1 AALS points per month, *p* = 0.015). In a logistic regression analysis, with progression rate as a binary outcome (fast progression versus slow progression), onset site was associated with progression rates (*p* = 0.019). In this analysis, differences between pairs were found with arm and leg onset sites as well as between bulbar and leg onset sites (arm vs. leg, odds ratio (OR) = 3.6, *p* = 0.016; bulbar vs. leg, OR = 6.4, *p* = 0.003).

**Figure 6:**
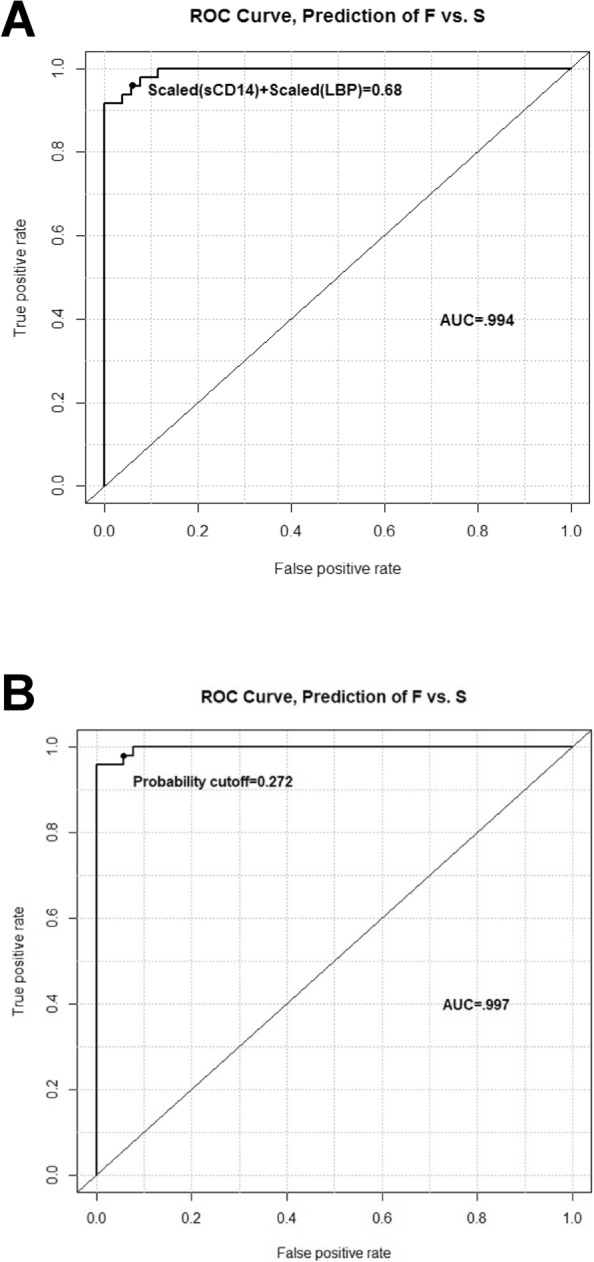
The addition of disease onset sites improves the predictive abilities of three APPs. (**A**) Serum sCD14 and LBP levels distinguish fast progressing patients from slowly progressing patients. The “probability cutoff” referenced in the graphic is the predicted probability of being in the fast progression group, according to the model equation, which is as follows: Probability of F vs. S = exp(− 4.618 + 5.622*[scaled(LBP) + scaled(sCD14)] + 0 (if site = A) − 1.934 (if site = L) + 1.747 (if site = B))**/**(1 + exp(− 0.618 + 5.622*[scaled(LBP) + scaled (sCD14)] + 0 (if site = A) − 1.934 (if site = L) + 1.747 (if site = B))). *Note* “Other” from this model was excluded because the data were too sparse to permit a reliable model. (**B**) Serum sCD14 levels, serum LBP levels, and disease onset sites distinguished fast progressing patients from slowly progressing patients.

Since onset site was associated with disease progression rates, the addition of disease onset site may improve the predictive abilities of the previously described three APPs. Thus, ROC analyses were performed using a scaled sCD14+ scaled LBP as the predictor (see figure legend for the equation describing the scaled APPs). In the second cohort of patients, scaled serum sCD14 and LBP levels were accurate indicators of disease progression rates. Using a scaled ROC cutoff of 0.68 µg/ml of control as positive, serum sCD14 and LBP levels had a 94% accuracy, 96% sensitivity and 93% specificity for distinguishing fast progressing patients from slowly progressing patients (Fig. 6A). Adding the sites of disease onset to the equation offered even better discrimination. Using a “predicted probability” cutoff of 0.27, a logistic regression model that included serum sCD14 levels, LBP levels and disease onset sites as predictors, had a 96% accuracy, 98% sensitivity and 96% specificity for distinguishing fast progressing patients from slowly progressing patients (Fig. 6B). A caveat to this last ROC curve analysis was that the serum sCD14+ LBP cutoff varied with disease onset site, so that it was not possible to have one cutoff value. Instead, a “predicted probability” cutoff was calculated.

### Discussion

Systemic inflammation is now recognized to play a prominent role in the pathobiology of ALS^1–6,28–31^. Previous studies have demonstrated that in both patients with ALS and animal models of ALS, as the disease progresses and the disease burden escalates, there is a concomitant increase in the profile of pro-inflammatory cytokines and APPs^3,16,28,29^. The responses of both adaptive and innate immune systems play pivotal and interdependent roles regulating the rate of disease progression and survival; Tregs, an adaptive immune response, were dysfunctional in patients with ALS and monocytes in these patients expressed a unique gene profile associated with pro-inflammatory immune responses, an innate immune response. The current report showed that serum sCD14, an APP, was increased and accurately predicted decreased survival times in patients. In accord with these observations, CD14^−/low^/CD16^+^ monocytes were decreased and the cell surface expression of CD14 on monocytes was decreased in patients, possibly through cleavage of mCD14 and adding to the pool of serum sCD14. More relevant is the observation that serum sCD14 was not elevated in patients with AD, FTD, PD, or CIDP.

In addition to sCD14, two other hepatic APPs, LBP and CRP, were also elevated in patients’ sera. An earlier study found a high incidence of mild liver dysfunction in patients with ALS; ultrastructural changes of hepatocytes with intrahepatic metabolic abnormalities were consistently found in patients with ALS^32^. A more recent study demonstrated that inflammation and oxidative stress, as-well-as the up-regulation of hepatic fibrosis-related proteins, induce liver dysfunction in transgenic ALS mice^33^. Thus, the present study provides evidence for a systemic pro-inflammatory innate immune response in the peripheral circulation of patients with ALS; this response is associated with increased disease burden and faster disease progression. In accord with sCD14, LBP and CRP were not elevated in patients with AD, FTD, PD, or CIDP. These elevated serum APPs, in combination with monocyte activation, reflect a pro-inflammatory environment that potentially further contributes to the deteriorating clinical status of patients with ALS.

The gut microbiome is a source of potentially disease-modifying bioactive metabolites, and these metabolites have recently been suggested to contribute to the pathogenesis of neurological disorders^34–36^. In this light, environmental factors such as low-molecular-mass metabolites that originate from the gastrointestinal tract may modulate metabolic, transcriptional and epigenetic programs^36,37^. A recent study found that altered gut microbiome worsens ALS symptoms in mice, possibly through intensifying a pro-inflammatory response^36^. Furthermore, in another recent study, it was found that α-synuclein could spread from the gut to brain via the vagus nerve in a mouse model of PD^38^. Thus, in both models, the gut microbiome may play a role in the inflammatory response.

CD14, a glycosylphosphatidylinositol (GPI)-anchored membrane glycoprotein, is a myeloid differentiation marker expressed by monocytes/macrophages and along with the Toll-like receptor-4 and MD-2, acts as a co-receptor for LPS. Monocyte activation-dependent shedding of mCD14 yields sCD14; this activation leads to enhanced production of various cytokines such as IL-1β and TNF-α14-16. LPS is a potent monocyte activator that binds to CD14 and induces cellular release of sCD14^20,39^. In recent years, many clinical studies focusing on sCD14 revealed that in infectious diseases, such as sepsis and HIV, sCD14 levels were elevated and associated with disease severity or worse prognosis^16^. The current report showed that sCD14 levels were increased in the serum of patients with ALS, but when separated into fast progressing and slowly progressing patients, sCD14 levels were only elevated in the serum of fast progressing patients. In addition, sCD14 levels were also elevated in the CSF of patients, but again, when separated into patients with different progression rates, sCD14 levels were only elevated in the CSF of fast progressing patients.

sCD14 is produced by hepatocytes, which represent the major source of APPs^14,40^. Interestingly, hepatocytes are also a major source of another APP, LBP. Therefore, LBP levels were also evaluated in the serum of patients with ALS. LBP was increased in the serum of all patients, but when the samples were separated into fast progressing and slowly progressing patients, LBP was also only elevated in the serum of fast progressing patients. Furthermore, serum LBP was positively correlated with the patient’s burden of disease. More intriguing, there was a positive correlation between LBP and sCD14 in these patients; as sCD14 increased there was a concomitant increase in LBP. A possible reason why free endotoxin/LPS was not increased in the serum of patients with ALS, in contrast to a previous report^41^, is that LPS is bound in the sCD14/LBP complex which may then be interacting with TLR-4 on the surface of cells not expressing mCD14, such as endothelial or epithelial cells, and thus exacerbating a pro-inflammatory response.

The data suggest that frequency of CD14^−/low^/CD16^+^ monocytes and cell surface expression of CD14 are reduced on monocytes from patients either by actively shedding CD14 from the cell surface or by the reduced production and subsequent expression of CD14 on the monocyte cell surface. In consideration of the evidence that there are decreased numbers of CD14^−/low^/CD16^+^ monocytes and decreased cell surface expression of mCD14 on CD14^+^/CD16^−^ and CD14^+^/CD16^+^ monocytes, the activation-dependent cleavage and shedding of mCD14 from monocytes may further contribute to the serum pool of sCD14. Since it is known that parenchymal microglia also express mCD14, a possible source of the elevated CSF sCD14 levels may be due to the shedding of mCD14 from activated microglia, in addition to the shedding mCD14 from activated monocytes/macrophages that have migrated into this compartment. Therefore, these data again support the concept of a pro-inflammatory myeloid response in patients with ALS, and especially in patients with fast progressing disease.

The regulation of TIM-3 during immune responses suggests divergent adaptive and innate immune functions^27^. TIM-3 molecule is also expressed on innate immune cells and acts synergistically with TLR signaling pathways to promote a pro-inflammatory response; TIM3 is considered a pro-inflammatory marker on monocytes and macrophages^26^. In contrast to the decrease in the numbers of CD14^−/low^/CD16^+^ monocytes from patients with ALS, the frequency of CD14^−/low^/CD16^+^/TIM-3^+^ monocytes was increased in both PBMC and isolated pan-monocytes from fast progressing patients. This is another indicator of the pro-inflammatory peripheral immune environment in these patients.

It is also known that human monocytes produce sCD14 by a protease-independent mechanism; sCD14 is released from intracellular pools of CD14. To determine if CD14 is actively produced in PBMC, and thus increasing the intracellular pool of CD14 and its possible release from these cells as sCD14, PBMC were assayed by qRT-PCR for CD14 mRNA levels. The CD14 mRNA was reduced in PBMC from patients, but when separated into fast progressing and slowly progressing patients, CD14 mRNA was only reduced in the fast progressing patients. Therefore, while sCD14 was increased in sera of fast progressing patients, CD14 mRNA from PBMC of these same patients was decreased, suggesting the increased serum levels of sCD14 were due to cleavage from monocyte surface and not due to the release of sCD14 from intracellular pools of CD14.

Several clinical studies have reported elevated serum levels of sCD14 in inflammatory conditions^14,42–47^. Despite the demonstration of increased inflammation associated with activated microglia/macrophages in AD and FTD, in this study, serum levels of sCD14 were not elevated in AD and FTD^48^. Furthermore, serum levels of sCD14 were not elevated in CIDP, an immune-mediated inflammatory neuropathy with increased T lymphocytes and macrophages and macrophage-mediated myelin stripping^49^. Thus, elevated levels of serum sCD14 are distinct for ALS and distinguish ALS from three other neurological diseases.

Süssmuth et al. evaluated sCD14 in the CSF of patients with ALS and found it to be reduced compared with controls^50^. In contrast, the current study found that sCD14 was elevated in the CSF of patients and was more profound when separated into rapidly from slowly progressing ALS patients; the more rapid the disease progression rate, the higher CSF level of sCD14. In addition, sCD14 was also elevated in the sera and urine of patients with ALS compared with HC; three disparate body fluids from patients had elevated levels of sCD14. These elevated sCD14 levels further correlated with disease progression rates and survival time; the more elevated levels of sCD14, the faster the progression rate and the shorter the survival time. In their discussion, Süssmuth et al. conclude that inflammation in the CNS and in the systemic circulation is suggested to be a key factor in the pathogenesis of ALS and that the immune system is protective early in the disease process but is destructive later in the process; slowly progressing patients often convert to rapidly progressing patients with a concomitant increase in pro-inflammatory immune profiles. The data presented in the current study corroborates these conclusions. However, when microglia/monocytes/macrophages are activated, mCD14 is cleaved from the surface of these cells; cleavage of CD14 from these cells is a known marker of innate immune system activation and inflammation. Thus, as serum/CSF/urine sCD14 increases, a more pro-inflammatory response is occurring which leads to faster progression rates, greater disease burden, and shorter survival times. In addition, what distinguishes the current study from the Süssmuth et al. report is that serum sCD14 was not elevated in patients with AD, FTD, PD, CIDP, neurological disorders with known pro-inflammatory CNS responses.

CRP is a classical APP regulated by pro-inflammatory cytokines and secreted by hepatocytes. A recent study of patients with ALS found that serum CRP was elevated in these patients; patients with high levels of CRP progressed faster than those with lower CRP levels^16^. In the current study, CRP was elevated in the fast progressing patients of the first cohort, but in the larger second cohort, CRP was elevated in both fast and slowly progressing patients. These data confirmed earlier reports, suggesting that the liver contributes to the pro-inflammatory response in ALS. In patients with other diseases associated with well-known pro-inflammatory responses such as Duchenne muscular dystrophy, type 2 diabetes, and peripheral neuropathy, elevated levels of CRP have been reported^51,52^. However, confirming a previous study, CRP was not elevated in patients with CIDP^53^.

ALS typically begins in the limbs, but about one third of cases are bulbar onset^1^. The current data confirmed that 25% patients with ALS had bulbar onset of disease. When this cohort was separated into fast and slowly progressing patients, 35% of the fast progressing patients had bulbar onset whereas 15% of the slowly progressing patients had bulbar onset. Furthermore, 75% of leg onset patients had slowly progressing disease while 25% of patients with fast progressing disease had leg onset. Conversely, 68% of the fast progressing patients had bulbar onset while 32% of the slowly progressing patients had bulbar onset. Thus clinically, leg onset was associated with slower rates of disease progression and prolonged survival while bulbar onset was associated with faster progression. These findings were consistent with previous clinical assessments suggesting that bulbar onset was associated with a faster progressing disease. In addition, the current data confirmed that patients with elevated sCD14 progressed faster to 100 AALS points and had a shorter life expectancy. Interestingly, age of first symptom and age at diagnosis were not associated with rates of disease progression. However, overall, disease onset site was associated with progression rates; the differences were between arm and leg onset sites as well as between bulbar and leg onset sites.

Serum levels of all three APPs, sCD14, LBP, and CRP, were accurate predictors of disease progression rates. Adding onset site to disease progression rates and APP levels provided potential clinical value with even better discrimination of accuracy, sensitivity, and specificity for distinguishing fast from slowly progressing disease. Levels of sCD14, LBP, and CRP showed a tendency to correlate with site of disease onset, but none reached statistical significance. Nevertheless, adding the onset sites to levels of APPs may well improve clinical trial design.

ALS is now considered a systemic multifactorial, multisystem disease in which inflammation and the immune system play important roles in disease development and disease progression^54^. In this context, production of APPs are possible indicators of such a response. The APR is critical to the body's ability to successfully respond to injury. It normally lasts only a few days; however, if continued unchecked, the APR may contribute to the development of chronic inflammatory states, tissue damage and disease^51^. Both acute and chronic inflammation stimulate the release of pro-inflammatory cytokines. Production of APPs is mainly regulated by IL-6, IL-1β, and TNF-α^14,55^. A recent meta-analysis reported that the levels of IL-6, IL-1β, and TNF-α, along with TNF receptor 1, IL-8, and vascular endothelial growth factor (VEGF), were increased in the blood of patients with ALS compared with HC subjects, suggesting a peripheral systemic pro-inflammatory response in these patients^8^. Another recent study demonstrated that ALS monocytes are skewed toward a pro-inflammatory state in the peripheral circulation and may play a role in ALS disease progression, especially in fast progressing patients^6^. In this study, 9 of the top 10 up-regulated differentially expressed genes were involved in inflammation with 7 of these 9 genes directly involved in pro-inflammatory pathways. Other studies have also reported that the pro-inflammatory cytokines TNF-α, IL-6, and IFN-γ were elevated in the plasma or serum samples of patients with ALS^56–58^. Thus, the current study supports the importance of inflammation in ALS with increased systemic levels of APPs as a direct indicator of this inflammation.

An earlier 3.5 year prospective study demonstrated that the adaptive immune system’s Tregs and Th2 lymphocytes accurately reflected a patient’s current progression rate and could be used to monitor disease progression and survival^3^. A later study established that Tregs from patients with ALS were dysfunctional and less effective in suppressing Tresp proliferation; the greater the Treg dysfunction the greater the clinically assessed disease burden and the faster the disease progression^5^. The present study indicates that increased serum sCD14 and LPB also accurately reflect the patient’s current progression rate and survival times, reinforcing the concept that both adaptive and innate immune responses contribute to the now recognized systemic inflammation in ALS.

The data reported in the current study showed that serum APPs were increased in patients with ALS and that these increased levels accurately reflect disease burden, progression rates, and survival times, and reinforce the concept of ALS as a disorder with extensive systemic pro-inflammatory responses. Furthermore, the elevation of these APPs was distinct for ALS and were not elevated in sera of patients with PD, dementia, and CIDP. However, there are several limitations to this study. First, while the number of serum and blood samples from patients with ALS was adequate, the number of samples obtained from patients with PD, dementia, and CIPD was limited. Therefore, increasing the number of patient samples for each disease would help draw more definitive interpretations between the levels of APPs and disease burdens both within each disease and also between diseases. Second, the data suggesting that the gut microbiome and liver may play a significant role in ALS disease pathoprogression is a relatively new concept. Nevertheless, this concept can be interwoven into the overall hypothesis that ALS is a disorder with extensive systemic pro-inflammatory responses; ALS now is considered a multifactorial, multisystem disease in which the CNS and peripheral immune systems play important roles in development and progression of disease. A third limitation to this study is that any attempt to modulate the pro-inflammatory responses in patients with ALS has been minimally successful or has failed. The reason is that all such immune drugs simultaneously suppress pro-inflammatory (Th1, Th17, and M1 myeloid cells) and anti-inflammatory (Tregs and M2 myeloid cells) arms of the immune system. By down regulating both arms, the ratio of pro- to anti-inflammatory arms is not changed, and pro-inflammatory activities still predominate. For this reason, we are actively treating patients with infusions of expanded Tregs in clinical trials and thus to increase the anti-inflammatory constituents and to change the pro- to anti-inflammatory ratio. The results of a Phase I trial have been encouraging^59^.

In light of these cross-sectional data supporting the specificity and sensitivity of APP levels as indices of disease burden, disease progression, and survival in ALS, the next question is whether longitudinal APP data accurately monitor disease burden and progression. More specifically as disease progresses, do levels of the APPs, sCD14, LBP, and CRP also increase? Do levels of APPs stabilize or even decrease in concert with clinical stabilization or improvement? Such data would be of considerable value in monitoring therapeutic efficacy in clinical trials. Finally, the increased sCD14 levels reflect increased pro-inflammatory activation of monocytes/macrophages/microglia in ALS patients, and CD14 on activated myeloid cells could represent a potentially significant therapeutic target.

It is well documented that there is a microglia-directed pro-inflammatory response in CNS tissues of patients with ALS^31,60–62^. Accordingly, the data presented in this report support the concept of an ongoing monocyte-directed pro-inflammatory environment that exists in the peripheral circulation of these patients, especially those patients with fast progressing disease^6^. The decreased numbers of CD14^−/low^/CD16^+^ monocytes, the decreased cell surface expression of mCD14 on CD14^+^/CD16^−^ and CD14^+^/CD16^+^ monocytes, and the increased percentage of CD14^−/low^/CD16^+^/TIM-3^+^ monocytes in these patients were in accord with this concept. This report demonstrates that peripheral APP responses are elevated in patients with ALS possibly due to altered gut microbiome and immune cells interacting with compromised peripheral axon terminals at the neuromuscular junctions outside the blood brain barrier (BBB) inducing tissue injury, as-well-as possibly due to neuroinflammatory responses at motor neurons within the BBB. More importantly, serum sCD14 levels were increased in fast progressing patients and accurately predicted decreased survival times. These data suggested that increased percentages of CD14^−/low^/CD16^+^/TIM-3^+^ monocytes were associated with increased rates of disease progression and augmented disease burden; increased serum sCD14 levels are indicative of monocyte activated mCD14 shedding, a direct measure of an enhanced innate immune response. Including the additional evidence that soluble LBP and CRP were also elevated in patients’ sera, these factors along with sCD14 constituted a collective immune profile that was distinct for ALS and further increased the specificity and sensitivity for the disease.

## Supplementary information

Supplementary Legends

Supplementary Figure S1.

Supplementary Figure S2.

## Data Availability

The datasets generated during and/or analyzed during the current study are available from the corresponding author on request.
