# Supplementary material for: Elevated acute phase proteins reflect peripheral inflammation and disease severity in patients with amyotrophic lateral sclerosis
Source: Sci Rep. 2020 Sep 17;10:15295. doi: 10.1038/s41598-020-72247-5 (PMC7499429; doi:10.1038/s41598-020-72247-5)
Supplement: Supplementary file 1 — Supplementary Legends [file 41598_2020_72247_MOESM1_ESM.docx]

**Supplemental figure legends**

**Supplemental figure 1.** sCD14 levels in patients with ALS, AD, FTD, PD, and CIDP. (**A**) Serum sCD14 levels were elevated in patients with ALS compared with serum from HC (**p* = 0.0001). (**B**) sCD14 was only elevated in serum from fast progressing patients compared with either slowly progressing patients (**p* < 0.001) or HC (**p* < 0.001). (**C**) CD14 mRNA was reduced in PBMC from patients with ALS compared with HC (**p* = 0.003). (**D**) CD14 mRNA was only reduced in the fast progressing patients compared with either slowly progressing (**p* < 0.001) patients or HC (**p* < 0.001). (**E**) There were no differences in the serum levels of sCD14 from patients with dementia compared with HC (*p* = 0.204). (**F**) There were no difference in sCD14 levels among patients with AD, MCI, or FTD. (**G**) Serum sCD14 levels were not different between patients with CIDP and HC. (**H**) Serum sCD14 levels in fast progressing patients with ALS were different from patients with CIDP; no differences were found between slowly progressing patients with ALS and patients with CIDP. There was no difference between patients with CIDP and the combined groups of HC. (**I**) Serum sCD14 levels were not different between patients with PD and HC. (**J**) Serum LBP levels were not different between patients with PD and HC. (**K**) Serum CRP levels were not different between patients with PD and HC.

**Supplemental figure 2.** Serum LBP and CRP is increased in the first cohort of patients with ALS. (**A**) LBP was increased in the sera of all patients compared with HC (**p* < 0.001). (**B**) LBP was elevated in fast (**p* < 0.001) and slowly (**p* < 0.001) progressing patients compared with HC. (**C**) CRP was elevated in the sera of all patients compared with HC (**p* = 0.008). (**D**) CRP was elevated in the fast progressing patients compared with either slowly (***p* < 0.048) progressing patients or HC (**p* = 0.006). (**E**) CRP was not elevated in patients with CIPD.
