# Supplementary figures and images for: Elevated acute phase proteins reflect peripheral inflammation and disease severity in patients with amyotrophic lateral sclerosis
Source: Sci Rep. 2020 Sep 17;10:15295. doi: 10.1038/s41598-020-72247-5 (PMC7499429; doi:10.1038/s41598-020-72247-5)

## Slide 1
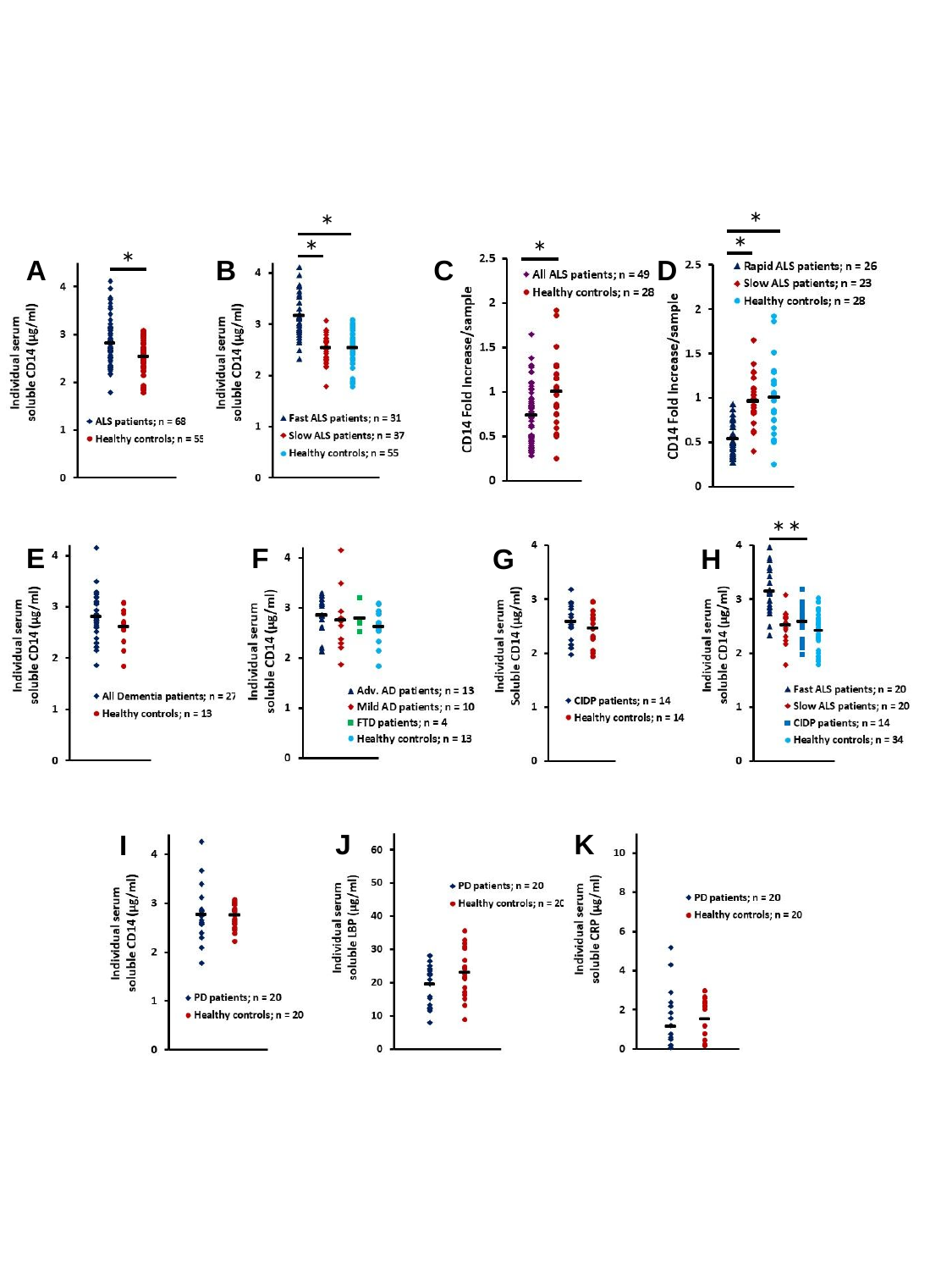

*
*
*
*
*
*
B
A
C
D
**
E
F
G
H
J
K
I

Supplement: Supplementary file 2 — Supplementary Figure S1. [file 41598_2020_72247_MOESM2_ESM.pptx]

## Slide 1
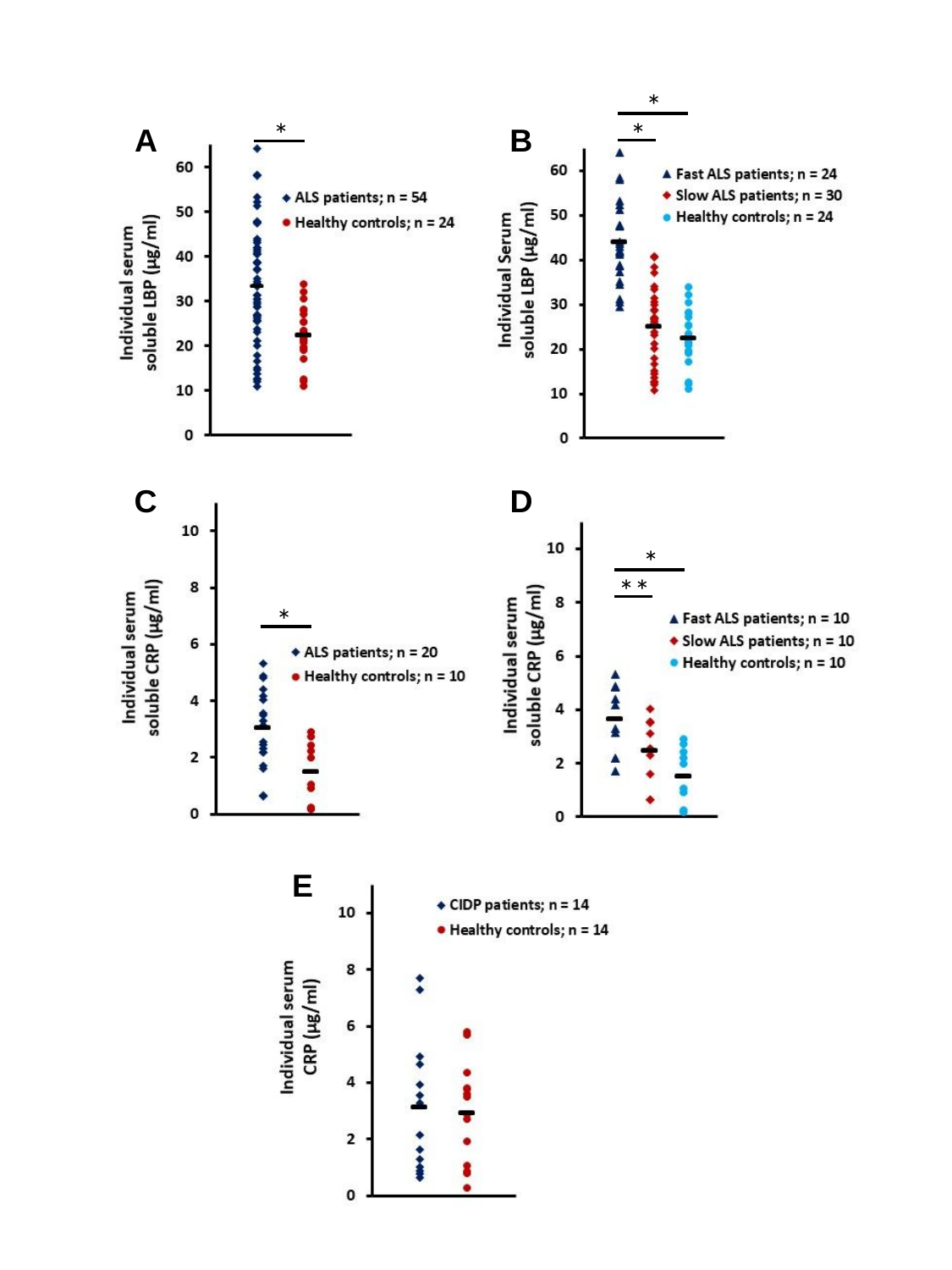

*
*
*
A
B
C
D
*
**
*
E

Supplement: Supplementary file 3 — Supplementary Figure S2. [file 41598_2020_72247_MOESM3_ESM.pptx]
